# ﻿Unveiling four new taxa and *Nigrosynnemanatarajanensis* comb. nov. in Stachybotryaceae (Hypocreales) from monocotyledon plants in Guangdong Province, China

**DOI:** 10.3897/mycokeys.114.139325

**Published:** 2025-03-05

**Authors:** Chunfang Liao, Mingkwan Doilom, D. Jayarama Bhat, Kandawatte Wedaralalage Thilini Chethana, Khanobporn Tangtrakulwanich, Yunhui Yang, Fatimah Al-Otibi, Kevin D. Hyde, Wei Dong

**Affiliations:** 1 Innovative Institute for Plant Health/Key Laboratory of Green Prevention and Control on Fruits and Vegetables in South China, Ministry of Agriculture and Rural Affairs, Zhongkai University of Agriculture and Engineering, Guangzhou 510225, China; 2 Center of Excellence in Fungal Research, Mae Fah Luang University, Chiang Rai 57100, Thailand; 3 School of Science, Mae Fah Luang University, Chiang Rai 57100, Thailand; 4 Vishnugupta Vishwavidyapeetam, Ashoke, Gokarna 581326, India; 5 Department of Botany and Microbiology, College of Science, King Saud University, P.O. Box 22452, Riyadh 11495, Saudi Arabia

**Keywords:** 4 new taxa, Asparagaceae, Hyphomycetes, saprobic fungi, Sordariomycetes, taxonomy, Zingiberaceae

## Abstract

Members of Stachybotryaceae are distributed worldwide, with certain species playing a significant role as bio-degraders and some causing diseases in plants, humans, and animals. Other species within this family can be found in soil and have been reported as saprobes in various plants. In this study (2021–2022), fungal taxa resembling Stachybotryaceae, isolated from dead leaves of *Agavesisalana* and a dead stem of *Wurfbainiavillosa* in Guangdong Province, China, are identified based on morphological characteristics and molecular data. Multi-locus phylogeny based on calmodulin (*cmdA*), internal transcribed spacer (ITS), the large subunit nuclear rDNA (LSU), RNA polymerase II second largest subunit (*rpb2*), the partial translation elongation factor 1-α (*tef1-α*), and β-tubulin (*tub2*) revealed that nine strains were grouped within Stachybotryaceae. *Nigrosynnemaguangdongense***gen. et sp. nov.**, typical of Stachybotryaceae but having unusual olivaceous brown to black synnemata that are narrower towards the apex and produce phialidic, aseptate, slimy conidia in black and glistening heads, is introduced. Additionally, *Brevistachyswurfbainiae* and *Sirastachysguangdongensis* are introduced as new species. *Stachybotrysmicrosporus* is a new host record for *Agavesisalana*. The present study provides comprehensive descriptions, illustrations, and molecular data analyses of the newly discovered taxa and newly recorded species as a taxonomic and phylogenetic contribution to Stachybotryaceae. Furthermore, a new combination, *Nigrosynnemanatarajanensis*, is proposed for the previously described *Virgatosporanatarajanensis*.

## ﻿Introduction

The family Stachybotryaceae (as Stachybotriaceae), belonging to Hypocreales, Sordariomycetes (Hyde et al. 2024), was established to accommodate the genera *Myrothecium*, *Peethambara*, and *Stachybotrys*, with *Stachybotrys* as the type (Crous et al. 2014). The members of this family are commonly isolated from soil and dead plant materials (Jie et al. 2012; Lombard et al. 2016; Hyde et al. 2017). Some species have been reported as pathogenic to plants and animals, with some posing a substantial risk to human health (Ben et al. 2015).

The polyphyletic nature of the genera *Myrothecium* and *Stachybotrys* was addressed by Lombard et al. (2016) through phylogenetic analyses using *cmdA*, ITS, LSU, *rpb2*, *tef1-α*, and *tub2*. As a result of their study, several species previously classified under *Myrothecium* and *Stachybotrys* were transferred into numerous other genera. Thirteen myrothecium-like genera were introduced, viz., *Albifimbria*, *Capitofimbria*, *Dimorphiseta*, *Gregatothecium*, *Inaequalispora*, *Myxospora*, *Neomyrothecium*, *Paramyrothecium*, *Parvothecium*, *Smaragdiniseta*, *Striaticonidium*, *Tangerinosporium*, and *Xenomyrothecium*. Eight stachybotrys-like genera were established, viz., *Achroiostachys*, *Brevistachys*, *Cymostachys*, *Globobotrys*, *Grandibotrys*, *Kastanostachys*, *Sirastachys*, and *Striatibotrys* (Lombard et al. 2016). In total, 33 genera were accommodated in the Stachybotryaceae, of which 21 were newly introduced and 20 were new combinations (Lombard et al. 2016). Additional novel genera were later introduced into this family by Hernandez-Restrepo et al. (2016), Gordillo and Decock (2017), Tibpromma et al. (2018), and Hyde et al. (2020). To date, 39 genera are accepted in this family (Hyde et al. 2020; Wijayawardene et al. 2022; Hyde et al. 2024). The family Stachybotryaceae is characterized by asexual morphs having mononematous, sporodochial, or synnematous conidiophores and phialidic conidiogenous cells that produce conidia in chains or in slimy masses (Crous et al. 2014; Wang et al. 2015; Lombard et al. 2016; Hyde et al. 2020). The sexual morph is described as having solitary ascomata, superficial or completely immersed in host tissue, bright to dark yellow, orange, or black that remain unchanged when treated with KOH, unitunicate asci rounded to nearly truncate at the apex with a refractive apical ring, and ellipsoidal to fusiform to broadly reniform ascospores (Subramanian and Bhat 1978; Crous et al. 2014; Lombard et al. 2016; Hyde et al. 2020).

The type genus *Stachybotrys* was introduced by Corda (1837) with *St.chartarum* as the type species. *Stachybotrys* shares a morphology similar to *Memnoniella* (introduced by Von Höhnel (1924), based on *Me.aterrima*) in having branched or unbranched, erect, thin-walled, smooth, or verrucous conidiophores (Bisby 1943; Wang et al. 2015). The conidia of *Memnoniella* are borne in dry chains, while those in *Stachybotrys* are produced in slimy masses. Smith (1962) considered both *Memnoniella* and *Stachybotrys* to be congeneric, arguing that the arrangement of conidia in dry chains (*Memnoniella*) or slimy masses (*Stachybotrys*) is insufficient for differentiating these two genera. In agreement with the argument provided by Smith (1962), Wang et al. (2015) synonymized *Memnoniella* under *Stachybotrys*. Phylogenetic analyses by Lombard et al. (2016), which included a broader sampling of taxa and more loci, clearly demonstrated that the isolate previously identified as *Memnoniellaechinata* (CBS 216.32) (*Me.aterrima*) by Galloway (1933) formed a distinct and well-supported clade separate from the *Stachybotrys* str clade. Therefore, Lombard et al. (2016) resurrected *Memnoniella* and designated the type species of the genus, *Me.Echinata*, as the epitype, using Galloway’s strain (Galloway 1933). Morphologically, *Memnoniella* can be distinguished from *Stachybotrys* by having mostly smooth, thick-walled, and unbranched conidiophores that give rise to linear dry chains of conidia (Lombard et al. 2016). The study by Lombard et al. (2016) was further supported by Lin et al. (2016), Doilom et al. (2017), Hyde et al. (2020), Mapook et al. (2020), Samarakoon et al. (2021), and Liu et al. (2024), all of which treated *Memnoniella* and *Stachybotrys* as distinct genera.

*Brevistachys*, introduced by Lombard et al. (2016) with *B.variabilis* as the type species, is characterized by conspicuously short conidiophores and conidiogenous cells that are borne either on conidiophores or directly from the vegetative hyphae, and obovoid to globose to ossiform to ellipsoidal conidia, aggregated in slimy masses. Five species are listed under *Brevistachys* in Index Fungorum (http://www.indexfungorum.org/names/names.asp; accessed on 12 October 2024). The asexual morph is only observed in *Brevistachys* species, which were isolated from *Musa* and *Zingiber* (Cooke 1883; Lombard et al. 2016). *Sirastachys*, introduced by Lombard et al. (2016), is typified by *Si.phaeospora*. The genus is characterized by cylindrical synnemata formed in culture, which consist of bundles of parallelly compacted, erect hyphae, with conidiophores arising laterally from synnemata. *Sirastachys* species were mainly isolated from leaves, with one species isolated from soil under *Thujaoccidentalis* (Lombard et al. 2016; Crous et al. 2018; Tibpromma et al. 2018). Nine species are listed under *Sirastachys* Fungorum (http://www.indexfungorum.org/names/names.asp; accessed on 25 July 2024).

In this study, we introduce one new genus, three new species, and one new host record in Stachybotryaceae from dead stems of *Wurfbainiavillosa* and a dead leaf of *Agavesisalana* in Guangdong Province, China, based on the morphological characteristics and multi-locus phylogenetic analyses of *cmdA*, ITS, LSU, *rpb2*, *tef1-α*, and *tub2*. The new taxa, *Brevistachyswurfbainiae* sp. nov., *Nigrosynnemaguangdongense* gen. et. sp. nov., and *Sirastachysguangdongensis* sp. nov., are compared to morphologically and phylogenetically closely related taxa. A new host record, *Stachybotrysmicrosporus*, is presented with a detailed description and illustration supported by phylogenetic evidence. Additionally, a new combination, *Nigrosynnemanatarajanensis* (= *Virgatosporanatarajanensis*), is proposed based on the similarity in morphological characteristics aligning with the generic concept of *Nigrosynnema* and supported by phylogenetic evidence of the type species of *Virgatospora*, *V.echinofibrosa*.

## ﻿Materials and methods

### ﻿Sample collection, morphological studies, and isolation

Samples of dead leaves of *Agavesisalana* and dead stems of *Wurfbainiavillosa* were collected in Guangdong Province, China, during the winter to spring seasons of 2021 and 2022, and the important collection information was noted (Rathnayaka et al. 2024). The morphological characteristics and microscopic examination of fungal structures were observed using the method described by Liao et al. (2023). Single spore isolation was performed following the methodology outlined in Senanayake et al. (2020). The living cultures were deposited in the Zhongkai University of Agriculture and Engineering Culture Collection (ZHKUCC), Guangdong, China. Specimens were deposited in the Mycological Herbarium of Zhongkai University of Agriculture and Engineering (MHZU), Guangzhou, China. The newly discovered species were registered in Faces of Fungi (FoF) (http://www.facesoffungi.org) (Jayasiri et al. 2015) and the Index Fungorum (IF) databases (http://www.indexfungorum.org/names/names.asp).

### ﻿DNA extraction, PCR amplification, and sequencing

The genomic DNA was extracted from the fungal mycelia cultivated in the dark at 25 °C on PDA for two weeks using the MagPure Plant DNA AS Kit, following the manufacturer’s instructions (Guangzhou Magen Biotechnology Co., Ltd, Guangdong, China). Extracted DNA was preserved at -20 °C for further molecular studies. The calmodulin (*cmdA*), internal transcribed spacer (ITS), large subunit rDNA (LSU), RNA polymerase II second largest subunit (*rpb2*), the partial translation elongation factor 1-α (*tef1-α*), and β-tubulin (*tub2*) were amplified and sequenced using primer CAL-228F (Carbone and Kohn 1999) and CAL-2RD (Groenewald et al. 2013), ITS4 and ITS5 (White et al. 1990), LR5 and LR0R (Vilgalys and Hester 1990), rpb2-5f and rpb2-7cR (Liu et al. 1999), EF1-728F (Carbone and Kohn 1999), EF2 (O’Donnell et al. 1998), and BT2a and BT2b (Glass and Donaldson 1995), respectively.

The polymerase chain reaction (PCR) contained a total of 25 µl of mixture, including 9.5 µl of ddH_2_O, 12.5 µl of 2 × Taq Master Mix (a mixture of dNTPs, optimized buffer, and Taq (Nanjing Vazyme Biotech Co., Ltd., Nanjing, China)), and 1 µl of each of the primer and DNA template. The PCR thermal cycling program for ITS and LSU amplification was conducted with an initial denaturation at 95 °C for 3 min, followed by 35 cycles of 94 °C for 30 sec; the annealing temperature was set to 52 °C for 30 sec; the extension step was performed at 72 °C for 1 min; and final elongation was carried out at 72 °C for 10 min. The annealing temperatures were altered to 53.5 °C for *cmdA* and *tef1-α* and 55 °C (45 sec) for *tub2*. PCR was performed for the *rpb2* in a thermal cycle as follows: an initial denaturation at 95 °C for 3 min, followed by 35 cycles at 94 °C for 1 min, an annealing temperature of 52 °C for 2 min, and extension at 72 °C for 1.5 min, with a final elongation at 72 °C for 10 min. The PCR products were purified and sent for sequencing at Tianyi Huiyuan Gene Technology & Services Co. (Guangdong, China). All sequences obtained in this study have been deposited in GenBank (available online: http://www.ncbi.nlm.nih.gov).

### ﻿Phylogenetic analyses

The original sequence obtained from the sequencing company was cross-checked by verifying chromatograms using BioEdit v. 7.2.3 (Hall 1999), and subsequently, consensus sequences were generated using OFPT (Zeng et al. 2023) and SeqMan v. 7.0 (Lasergene, Madison, WI, USA). The consensus sequences of our fungal strains from each locus were subjected to a basic local alignment search tool (BLAST) search in GenBank. The reference sequences and outgroup taxa used for phylogenetic analyses were selected based on recent relevant literature (Lombard et al. 2016; Tibpromma et al. 2018; Mapook et al. 2020), obtained from GenBank. The phylogenetic analyses utilized 184 sequences (Table 1), with *Fusariumsambucinum* strains CBS 146.95 and CBS 136.24 used as the outgroup taxa. Six loci, *cmdA*, ITS, LSU, *rpb2*, *tef1-α*, and *tub2*, were aligned using the MAFFT version v. 7 online program (https://mafft.cbrc.jp/alignment/server/; Katoh and Standley 2013). Subsequently, the dataset was trimmed with TrimAl v.1.3 using the Gappyout option (Capella-Gutiérrez et al. 2009). The alignments were transformed into NEXUS format using the ALignment Transformation EnviRonment online platform (http://www.sing-group.org/ALTER/).

**Table 1.** Names, culture collection numbers, and corresponding GenBank accession numbers of the taxa used in the phylogenetic analyses. The new strains in this study are indicated in cells with light blue shading. “^T^” is used to represent ex-type. “-” denotes unavailable information.

Taxa	Culture collection numbers	GenBank accession numbers					
		*cmdA*	ITS	LSU	*rpb2*	*tef1-α*	*tub2*
*Achroiostachyshumicola*	CBS 317.72	KU845777	KU845797	KU845817	KU845835	KU845852	KU845758
*A.humicola*	CBS 868.73^T^	KU845779	KU845799	KU845819	KU845837	KU845854	KU845760
*A.levigata*	CBS 185.79^T^	KU845785	KU845805	KU845825	KU845841	KU845860	KU845765
*A.levigata*	CBS 363.58	KU845786	KU845806	KU845826	KU845842	KU845861	KU845766
*Albosynnemaelegans*	GB 3101^T^	-	-	AF193226	-	-	-
*Albifimbriaverrucaria*	CPC 30056	KU845869	KU845885	KU845904	KU845923	KU845942	KU845961
*Al.verrucaria*	CBS 176.27	KU845870	KU845886	KU845905	KU845924	KU845943	KU845962
*A..viridis*	CBS 244.78	-	KU845897	KU845916	KU845935	KU845954	KU845973
*A..viridis*	CBS 449.71^T^	KU845879	KU845898	KU845917	KU845936	KU845955	KU845974
*Alfariacaricicola*	CBS 113567^T^	KU845976	KU845983	KU845992	KU846001	KU846008	KU846014
*A.f.cyperiesculenti*	CPC 23153^T^	-	KJ869143	KJ869200	-	-	-
*A.f.thymi*	CBS 447.83^T^	KU845981	KU845990	KU845999	-	KU846013	KU846021
*Alfariacladiellaspartii*	CPC 24966	-	NR_164243	NG_070399	-	-	-
*Brevistachysglobosa*	CBS 141056^T^	KU846024	KU846038	KU846057	-	KU846085	KU846101
*B.globosa*	CPC 15952	KU846025	KU846040	KU846059	-	KU846087	KU846103
*B.globosa*	CPC 16060	-	KU846042	KU846061	-	KU846089	KU846105
*B.lateralis*	CBS 141058^T^	KU846027	KU846043	KU846062	KU846074	KU846090	KU846106
*B.ossiformis*	CBS 696.73^T^	-	KU846044	KU846063	-	-	KU846107
*B.ossiformis*	CBS 112792	KU846028	KU846045	KU846064	KU846075	KU846091	KU846108
*B.ossiformis*	CPC 16031	KU846029	KU846046	KU846065	-	KU846092	KU846109
*B.subsimplex*	ATCC 32888^T^	-	AF205439	-	-	-	-
*B.variabilis*	CBS 141057	KU846030	KU846047	KU846066	KU846076	KU846093	KU846110
*B.wurfbainiae*	ZHKUCC 23-1011^T^	PP746514	PP645738	PP683135	PP746507	PP746523	PP746532
*B.wurfbainiae*	ZHKUCC 23-1012	PP746515	PP645739	PP683136	PP746508	PP746524	PP746533
*B.wurfbainiae*	ZHKUCC 23-1013	PP746516	PP645740	PP683137	PP746509	PP746525	PP746534
*Capitofimbriacompacta*	CBS 111739^T^	KU846261	KU846287	KU846317	KU846349	KU846378	KU846404
*C.compacta*	MUCL 50238	-	KU878556	KU878557	KU878558	-	KU878559
*Cymostachyscoffeicola*	CBS 252.76^T^	KU846035	KU846052	KU846071	KU846081	KU846097	KU846113
*Cy.coffeicola*	CPC 25009	-	KU846053	-	-	-	-
*Cy.fabispora*	CBS 136180^T^	KU846036	KU846054	KU846072	KU846082	KU846098	KU846114
*Cy.fabispora*	CPC 24352	-	KU846055	-	KU846083	KU846099	-
*Didymostilbeaurantispora*	CBS 616.85^T^	-	-	KU846344	-	-	-
*D.matsushimae*	CBS 549.84	-	-	KU846345	-	-	-
*D.matsushimae*	CCFC 54984	-	-	AY283545	-	-	-
*Digitisetadimorpha*	MUCL 54683^T^	-	KY389329	KY389349	KY389367	KY769935	KY366460
*D..multidigitata*	MUCL 41187^T^	-	KY389325	KY389345	KY389363	KY769934	KY366456
*D..parvidigitata*	MUCL 48180^T^	-	KY389326	KY389346	KY389364	KY769931	KY366457
*D..parvidigitata*	MUCL 48271	-	KY389327	KY389347	KY389365	KY769932	KY366458
*D..parvidigitata*	MUCL 48260	-	KY389328	KY389348	KY389366	KY769933	KY366459
*D..setiramosa*	CBS 534.88	-	AY254156	-	-	KY769930	-
*Dimorphisetaterrestris*	CBS 127345^T^	KU846284	KU846314	KU846346	KU846375	KU846401	KU846431
*Fusariumsambucinum*	CBS 146.95	-	-	KM231682	KM232381	-	-
*F.sambucinum*	CBS 136.24	-	-	MH866281	-	-	-
*Globobotryssansevieriicola*	CBS 138872^T^	-	KR476717	KR476752	-	KR476793	KR476794
*Grandibotryspseudotheobromae*	CBS 136170^T^	-	KU846135	KU846161	KU846188	KU846215	KU846241
*G.pseudotheobromae*	CBS 136391	-	KU846136	KU846162	KU846189	KU846216	KU846242
*G.xylophila*	CBS 136179^T^	KU846115	KU846137	KU846163	KU846190	KU846217	
*Gregatotheciumhumicola*	CBS 205.96^T^	KU846285	KU846315	KU846347	KU846376	KU846402	KU846432
*Hyalinostachyscylindrospora*	MFLUCC 17-2583	-	NR_182450	NG_148956	-	-	-
*Inaequalisporaprestonii*	CBS 175.73^T^	KU846286	KU846316	KU846348	KU846377	KU846403	KU846433
*Kastanostachysaterrima*	CBS 101310^T^	-	-	AF178565	KU846191	-	-
*Koorchalomabambusae*	MFLU 19-2899	-	MT185516	MT183479	MT432230	-	-
*K.europaea*	PRM 953076	-	LR963471	-	-	-	-
*K.krabiense*	MFLUCC 16-0317^T^	-	MH388348	MH376721	MH412729	-	-
*K.oryzae*	MFLUCC 21-0055^T^	-	MZ519544	MZ519543	MZ508427	-	-
*K.spartinicola*	SAP 130	-	AF422963	-	-	-	-
*Koorchalomellasalmonispora*	MD6018	-	-	KX611345	-	-	-
*Melanopsammapomiformis*	CBS 325.90	KU846031	KU846048	KU846067	KU846077	KU846094	KU846111
*M.pomiformis*	CBS 101322^T^	KU846032	KU846049	KU846068	KU846078	-	-
*M.pomiformis*	CBS 114119	KU846033	KU846050	KU846069	KU846079	KU846095	KU846112
*M.xylophila*	CBS 100343^T^	KU846034	KU846051	KU846070	KU846080	KU846096	-
*Memnoniellaalishanensis*	MFLUCC 20-0168^T^	-	MW114372	-	-	-	MW148278
*Me.brunneoconidiophora*	CBS 109477	-	KU846138	KU846165	KU846192	KU846218	KU846243
*Me.brunneoconidiophora*	CBS 136191^T^	KU846116	KU846139	KU846166	KU846193	KU846219	KU846244
*Me.celtidis*	MFLUCC 20-0040^T^	-	MW114374	-	-	-	MW148280
*Me.celtidis*	NCYUCC 19-0326	-	MW114375	-	-	-	MW148281
*Me.chromolaenae*	MFLUCC 17-1507	-	NR_168873	MT214465	-	-	-
*Me.dichroa*	CBS 526.50	KU846117	KU846140	KU846167	KU846194	KU846220	-
*Me.dichroa*	CBS 123800	KU846118	KU846141	KU846168	KU846195	KU846221	-
*Me.echinata*	CBS 216.32^T^	KU846119	KU846142	KU846169	KU846196	KU846222	KU846245
*Me.echinata*	DAOMC 173162	KU846125	JN942886	JN938868	KU846202	KU846228	KU846250
*Me.echinata*	DAOMC 235365	KU846126	KU846149	KU846176	KU846203	KU846229	KU846251
*Me.ellipsoidea*	CBS 136199	KU846127	KU846150	KU846177	KU846204	KU846230	KU846252
*Me.ellipsoidea*	CBS 136200	KU846128	KU846151	KU846178	KU846205	KU846231	KU846253
*Me.ellipsoidea*	CBS 136201^T^	KU846129	KU846152	KU846179	KU846206	KU846232	KU846254
*Me.humicola*	CBS 463.74^T^	KU846130	KU846154	KU846181	KU846208	KU846234	
*Me.longistipitata*	CBS 136197	KU846131	KU846155	KU846182	KU846209	KU846235	KU846256
*Me.longistipitata*	ATCC 22699	-	AF081471	-	-	-	-
*Me.mori*	MFLUCC 18-1640^T^	-	MW114377	-	-	-	MW148283
*Me.nilagirica*	MFLUCC 15-0660	-	KU760374	-	KU760394	-	-
*Me.oblongispora*	MFLUCC 17-2064	-	MT310665	-	MT394724	-	-
*Me.oblongispora*	MFLUCC 15-1074	KY124123	KU760376	-	KU760396		KY124127
*Me.oenanthes*	ATCC 22844^T^	-	AF081473	-	-	-	-
*Me.oenanthes*	CBS 388.73	-	KU846156	KU846183	KU846210	KU846236	
*Me.Pseudodichroa*	BCRC FU31689^T^	-	ON692522	-	LC714856	LC714858	LC714861
*Me.Pseudodichroa*	BCRC FU31700	-	ON692523	-	LC714857	LC714859	LC714862
*Me.pseudonilagirica*	CBS 136405^T^	KU846132	KU846157	KU846184	KU846211	KU846237	KU846257
*Me.putrefolia*	CBS 101177^T^	-	KU846158	KU846185	KU846212	KU846238	KU846258
*Me.putrefolia*	CBS 136171	KU846133	KU846159	KU846186	KU846213	KU846239	KU846259
*Me.sinensis*	YMF 1.05582^T^	-	MK773576	-	MK773575	MK772066	MK773574
*Memnoniella* sp.	MUCL 50191	KU846134	KU846160	KU846187	KU846214	KU846240	KU846260
*Myrotheciuminundatum*	CBS 196.74	KU846434	KU846451	KU846473	-	KU846513	KU846532
*My.inundatum*	CBS 275.48^T^	KU846435	KU846452	KU846474	-	KU846514	KU846533
*My.inundatum*	CBS 120646	KU846438	KU846455	KU846477	-	KU846516	KU846536
*My.simplex*	CBS 582.93^T^	KU846439	KU846456	KU846478	-	KU846517	KU846537
*Myxosporacrassiseta*	CBS 731.83^T^	KU846442	KU846459	KU846481	KU846497	KU846520	KU846540
*Myx.crassiseta*	CBS 121141	KU846443	KU846460	KU846482	KU846498	KU846521	KU846541
*Myx.masonii*	CBS 174.73^T^	KU846445	KU846462	KU846484	KU846500	KU846523	KU846543
*Neomyrotheciumhumicola*	CBS 310.96^T^	KU846448	KU846467	KU846488	KU846505	KU846527	-
*Nigrosynnemaguangdongense*	ZHKUCC 23-1014^T^	PP746517	PP645741	PP683138	PP668100	PP746526	PP746535
*N.guangdongense*	ZHKUCC 23-1015	PP746518	PP645742	PP683139	PP668101	PP746527	PP746536
*Paramyrotheciumfoliicola*	CBS 113121^T^	KU846266	KU846294	KU846324	-	KU846385	KU846411
*P.roridum*	CBS 212.95	KU846269	KU846299	KU846329	KU846360	KU846389	KU846416
*P.roridum*	CBS 357.89^T^	KU846270	KU846300	KU846330	KU846361	KU846390	KU846417
*Parvotheciumterrestre*	CBS 198.89^T^	KU846449	KU846468	KU846489	KU846506	KU846528	KU846548
*Parvotheciumterrestre*	CBS 534.88	KU846450	KU846469	KU846490	KU846507	KU846529	KU846549
*Peethambarasundara*	CBS 521.96	-	KU846470	KU846491	KU846508	KU846530	KU846550
*Pe.sundara*	CBS 646.77^T^	-	KU846471	AF193245	KU846509	KU846531	KU846551
*Septomyrotheciummaraitiense*	MUCL 47202^T^	-	-	KU846493	KU846510	-	-
*S.uniseptatum*	CBS 100966	-	KU846472	KU846494	KU846511	-	KU846552
*S.uniseptatum*	MUCL 52944	-	-	KU846495	KU846512	-	-
*Sirastachyscastanedae*	CBS 164.97	KU846553	KU846658	KU846771	KU846885	KU846990	KU847094
*Si.castanedae*	CBS 531.69	KU846554	KU846659	KU846772	KU846886	KU846991	KU847095
*Si.castanedae*	CBS 136403^T^	KU846555	KU846660	KU846773	KU846887	KU846992	KU847096
*Si.castanedae*	CPC 20373	KU846556	KU846661	KU846774	KU846888	KU846993	KU847097
*Si.cylindrospora*	CBS 136166^T^	KU846557	KU846662	KU846775	KU846889	-	KU847098
*Si.cylindrospora*	CBS 13654	KU846558	KU846663	KU846776	KU846890	KU846994	KU847099
*Si.cyperacearum*	CBS 143444	-	MH107917	MH107963	-	-	-
*Si.guangdongensis*	ZHKUCC 23-1003^T^	PP746510	PP645734	PP683131	PP754606	PP746519	PP746528
*Si.guangdongensis*	ZHKUCC 23-1004	PP746511	PP645735	PP683132	PP754607	PP746520	PP746529
*Si.longispora*	ATCC 32451^T^	-	AF081482		-	-	-
*Si.pandanicola*	CBS 136545^T^	-	KU846664	KU846777	-	-	KU847100
*Si.phaeospora*	CBS 100155^T^	KU846560	KU846666	KU846779	KU846891	KU846995	KU847102
*Si.phangngaensis*	MFLUCC 15-0680	-	NR_168202	NG_068841	MH412735	-	-
*Si.phyllophila*	CBS 173.97	KU846565	KU846671	KU846784	KU846896	KU846998	KU847107
*Si.phyllophila*	CBS 136169^T^	KU846566	KU846672	KU846785	KU846897	KU846999	KU847108
*Si.pseudolongispora*	CBS 417.93	KU846567	KU846673	KU846786	KU846898	KU847000	KU847109
*Si.pseudolongispora*	CBS 100154^T^	KU846568	KU846674	KU846787	KU846899	-	KU847110
*Sirastachys* sp.	CBS 308.56	KU846569	KU846675	KU846788	KU846900	KU847001	KU847111
*Smaragdinisetabisetosa*	CBS 459.82^T^	KU847206	KU847229	KU847255	KU847281	KU847303	KU847319
*Stachybotrysaloeticola*	CBS 137940^T^	KU846570	KJ817888	KJ817890	KU846901	-	KJ817886
*St.aloeticola*	CBS 137941	KU846571	KJ817889	KJ817891	KU846902	-	KJ817887
*St.chartarum*	CBS 182.80^T^	KU846573	KU846679	KU846792	KU846904	KU847003	KU847115
*St.chartarum*	CBS 119371	KU846594	KU846700	KU846813	KU846925	KU847024	KU847135
*St.chartarum*	CBS 485.48	KU846577	KU846683	KU846796	KU846908	KU847007	KU847119
*St.chlorohalonata*	CBS 113.97	KU846635	KU846742	KU846855	KU846965	KU847065	KU847176
*St.chlorohalonata*	CBS 127.94	KU846636	KU846743	KU846856	KU846966	KU847066	KU847177
*St.chlorohalonata*	CBS 222.46	KU846637	KU846744	KU846857	KU846967	KU847067	KU847178
*St.chlorohalonata*	CBS 250.89	KU846617	KU846723	KU846836	KU846948	KU847047	KU847158
*St.chlorohalonata*	CBS 109283	KU846622	KU846728	KU846841	KU846953	KU847052	KU847163
*St.chlorohalonata*	CBS 109285^T^	KU846623	KU846729	KU846842	KU846954	KU847053	KU847164
*St.chlorohalonata*	CBS 136158	KU846626	KU846732	KU846845	KU846956	KU847056	KU847167
*St.dolichophialis*	DAOMC 227011	KU846628	KU846734	KU846847	KU846958	-	KU847169
*St.limonispora*	CBS 128809^T^	KU846629	KU846735	KU846848	KU846959	KU847058	KU847170
*St.limonispora*	CBS 136165	KU846630	KU846736	KU846849	KU846960	KU847059	KU847171
*St.microsporus*	CBS 186.79	KU846631	KU846737	KU846850	DQ676580	KU847060	KU847172
*St.microsporus*	ATCC 18852^T^	-	AF081475	-	-	-	-
*St.microsporus*	MFLUCC 15-0830	KY124124	KU760377	-	KU760397	-	KY124128
*St.microsporus*	MFLUCC 15-1076	KY124125	KU760378	-	KU760398	-	KY124129
*St.microsporus*	MFLUCC 20-0190	-	MW477992	-	-	MW480237	MW480235
*St.microsporus*	ZHKUCC 23-1007	PP746512	PP645736	PP683133	PP668098	PP746521	PP746530
*St.microsporus*	ZHKUCC 23-1008	PP746513	PP645737	PP683134	PP668099	PP746522	PP746531
*St.musae*	MFLUCC 20-0188^T^	MW480232	NR_173231	-	MW480230	-	MW480234
*St.musae*	MFLUCC 20-0152	MW480231	MW477991	-	MW480229	-	MW480233
*St.pallescens*	HGUP 0146^T^		KC305345	KC305345	-	-	-
*St.phaeophialis*	KAS 525^T^	KU846632	KU846738	KU846851	KU846962	KU847061	KU847173
*St.reniformis*	CBS 976.95	KU846633	KU846739	KU846852	KU846963	KU847062	KU847174
*St.reniformis*	CBS 136198	-	KU846740	KU846853	-	KU847063	-
*St.subcylindrospora*	HGUP 0201^T^	-	KC305354	-	-	-	-
*St.subreniformis*	HGUP 1051^T^	-	KC305344	-	-	-	-
*St.subsylvatica*	CBS 126205^T^	KU846634	KU846741	KU846854	KU846964	KU847064	KU847175
*Stachybotrys* sp.	CBS 525.50	KU846645	KU846752	KU846865		KU847075	KU847186
*Striatibotryseucylindrospora*	CBS 203.61^T^	KU846648	KU846755	KU846868	KU846975	KU847078	KU847189
*Str.eucylindrospora*	CBS 136399	-	KU846757	KU846870	KU846977	KU847080	KU847191
*Str.eucylindrospora*	CBS 136547	KU846649	KU846758	KU846871	KU846978	KU847081	KU847192
*Str.neoeucylindrosporus*	UAMH 7211	-	MW187767	MW187732	MW192603	MW192605	MW192606
*Str.neoeucylindrosporus*	UAMH 7122	-	MW187766	MW187768	MW192608	MW192609	MW192610
*Striaticonidiumbrachysporum*	CBS 131.71	KU847207	KU847230	KU847256	KU847282	KU847304	KU847320
*Stri.brachysporum*	CBS 513.71^T^	KU847209	KU847232	KU847258	KU847284	KU847305	KU847322
*Stri.brachysporum*	CBS 126552	KU847210	KU847233	KU847259	KU847285	KU847306	KU847323
*Stri.cinctum*	CBS 373.50	KU847214	KU847237	KU847263	KU847289	-	KU847327
*Stri.cinctum*	CBS 932.69^T^	KU847216	KU847239	KU847265	KU847290	-	KU847329
*Stri.Deklijnearum*	CBS 143232	-	NR_156676	NG_058527	-	MG386158	MG386171
*Stri.humicola*	CBS 258.76^T^	-	KU847240	KU847266		KU847311	KU847330
*Stri.humicola*	CBS 388.97	KU847217	KU847241	KU847267	KU847291	KU847312	KU847331
*Stri.synnematum*	CBS 479.85^T^	KU847218	KU847242	KU847268	KU847292	-	KU847332
*Tangerinosporiumthalitricola*	CBS 317.61^T^	KU847219	KU847243	KU847269	-	-	KU847333
*Virgatosporaechinofibrosa*	CBS 110115	KU847220	KU847244	KU847270	KU847293	KU847313	KU847334
*V.echinofibrosa*	MUCL 39092	-	KU847245	KU847271	KU847294	-	KU847335
*Xenomyrotheciumtongaense*	CBS 598.80^T^	KU847221	KU847246	KU847272	KU847295	KU847314	KU847336
*Xepiculajollymannii*	CBS 276.48^T^	KU847223	KU847248	KU847274	KU847297	KU847316	KU847338
*X.jollymannii*	CBS 126168	KU847224	KU847250	KU847276	KU847298	KU847317	KU847340
*X.leucotricha*	CBS 278.78	KU847227	KU847253	KU847279	KU847301	-	KU847343
*X.leucotricha*	CBS 483.78	KU847228	KU847254	KU847280	KU847302	KU847318	KU847344

The combined *cmdA*, ITS, LSU, *rpb2*, *tef1-α*, and *tub2* sequence data were performed using maximum likelihood (ML) and Bayesian inference (BI) analyses. The ML analysis was carried out in the CIPRES Science Gateway online platform (Miller et al. 2010) using RAxMLHPC v.8.2.12 on XSEDE (Stamatakis 2014) with GTR+G+I evolutionary substitution, with 1000 rapid bootstrap inferences followed by an extensive ML search. All free model parameters were estimated using the RAxML maximum likelihood method with 25 per-site rate categories. The likelihood of the final tree was evaluated and optimized under the GAMMA gamma distribution shape parameter. The Bayesian Inference (BI) analysis was conducted utilizing the Markov Chain Monte Carlo (MCMC) method and executed in MrBayes XSEDE (3.2.7a) (Huelsenbeck and Ronquist 2001). The simulation was conducted by running six concurrent Markov chains for 5,000,000 generations, with tree sampling occurring every 100^th^ generation. The phylogenetic trees were visualized using FigTree v. 1.4.0 (Rambaut 2009) and formatted using PowerPoint 2010 (Microsoft Corporation, WA, United States). New species are established based on the recommendations of Jeewon and Hyde (2016).

## ﻿Results

### ﻿Phylogenetic analyses

**Figure 1.**
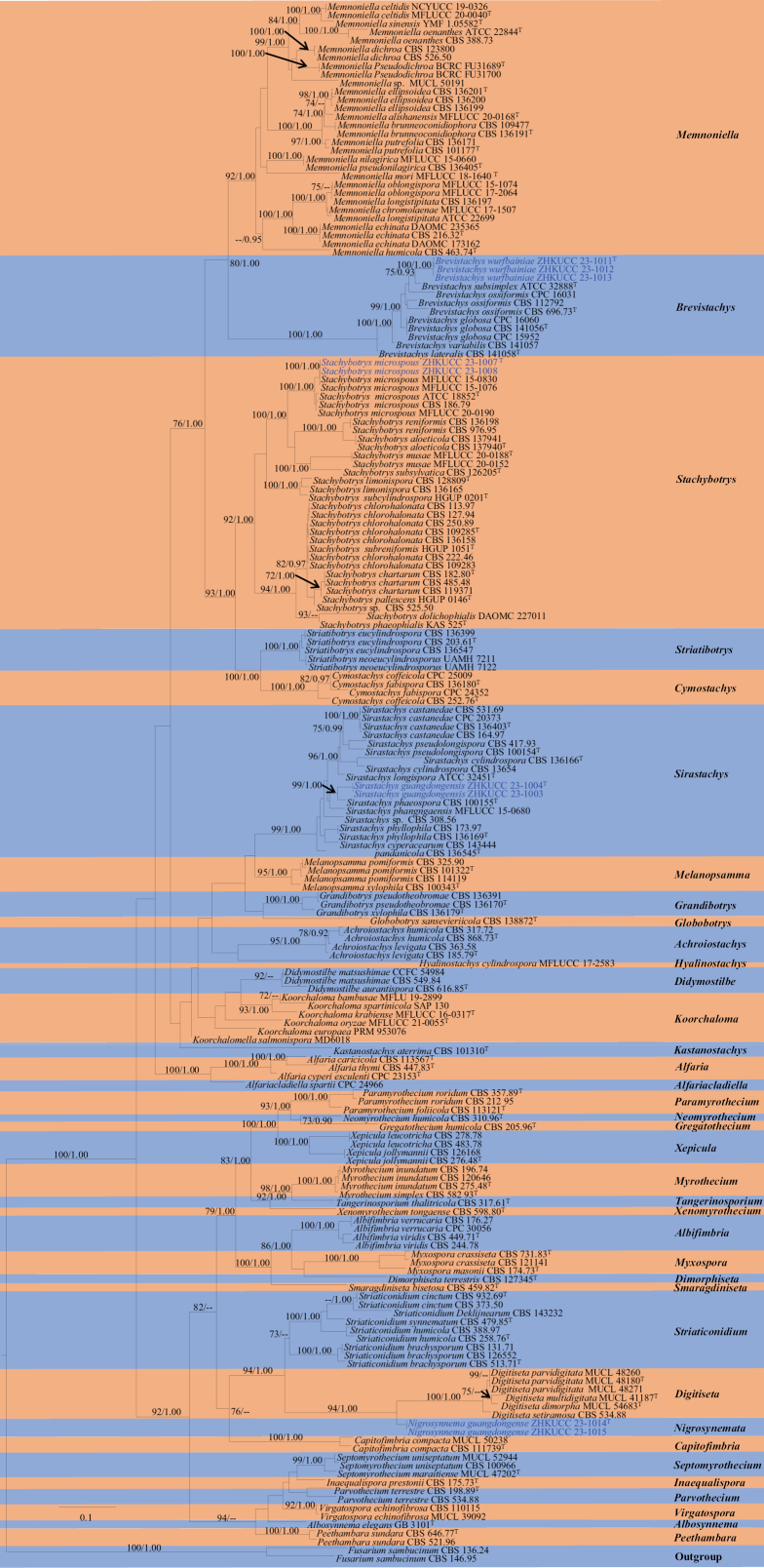
Phylogram generated from maximum likelihood analysis (RAxML) of strains in Stachybotryaceae based on the combined *cmdA*, ITS, LSU, *rpb2*, *tef1-α*, and *tub2* sequence data. Maximum likelihood bootstrap values ≥ 70% (ML) and Bayesian posterior probabilities ≥ 0.90 (ML/BYPP) are provided at the nodes. The tree is rooted with *Fusariumsambucinum* strains CBS 136.24 and CBS 146.95. The hyphen (-) represents support values < 70% ML and < 0.90 BYPP. The ex-type strains are denoted as “^T^”, while the newly isolated strains are highlighted in blue.

The phylogenetic tree was constructed using the combined *cmdA*, ITS, LSU, *rpb2*, *tef1-α*, and *tub2* sequence data of 184 strains (including our new strains) through ML and BI analyses. The total length of the dataset, including gaps, was 5060 base pairs (*cmdA*: 1–930, ITS: 931–1657, LSU: 1658–2494, *rpb2*: 2495–3274, *tef1*-α: 3275–4646, *tub2*: 4647–5060). The topology of the ML analysis resembled that of the BI analysis. The highest-scoring RAxML tree, with a final ML optimization likelihood value of -87901.129587, is depicted in Fig. 1. The matrix consisted of 1060 distinct alignment patterns, with 44.52% undetermined characters or gaps. The estimated base frequencies were as follows: A = 0.235675, C = 0.272976, G = 0.268686, T = 0.222663; substitution rates AC = 1.088667, AG = 3.185610, AT = 1.213744, CG = 0.825063, CT = 4.778952, GT = 1.000000; gamma distribution shape parameter α = 0.307577. In this study, the phylogenetic analyses showed that our strains belong to Stachybotryaceae. The tree topology in this study is almost similar to the previous studies of Lombard et al. (2016) and Hyde et al. (2020). However, Lombard et al. (2016) and Hyde et al. (2020) constructed the tree using LSU and *rpb2*. The inclusion of an increasing number of newly discovered genera and taxa, including *Digitiseta* gen. nov. and additions to *Inaequalispora* and *Parvothecium* as reported by Gordillo and Decock (2017), has resulted in slight alterations to the positions of some genera. Additionally, our phylogenetic tree showed that species of *Koorchaloma* are paraphyletic and grouped with *Didymostilbe* instead of *Koorchalomella* and *Alfariacladiella* as shown in Hyde et al. (2020). Two new strains (ZHKUCC 23-1007, ZHKUCC 23-1008) constituted a highly supported subclade with *Stachybotrysmicrosporus* (type strain, CBS 126205) with 100% ML and 1.00 BYPP. The novel strains ZHKUCC 23-1003 and ZHKUCC 23-1004 formed a sister subclade with *Sirastachysphaeospora* (type strain, CBS 100155) with 99% ML bootstrap support and 1.00 BYPP. Three strains, ZHKUCC 23-1011, ZHKUCC 23-1012, and ZHKUCC 23-1013, formed a sister subclade with *Brevistachyssubsimplex* (type strain, ATCC 32888) with 75% ML and 0.93 BYPP. Two strains, ZHKUCC 23-1014 and ZHKUCC 23-1015, formed a distinct clade with *Digitiseta* species with 94% ML and 1.00 BYPP.

### ﻿Taxonomy

#### ﻿ Brevistachys wurfbainiae

Taxon classificationFungiHypocrealesStachybotryaceae

C.F. Liao, K.D. Hyde & Doilom sp. nov.

B7ABE979-3743-55CB-BBC2-ECDDF53A6B59

Index Fungorum: IF902005

Facesoffungi Number: FoF15745

Fig. 2

##### Etymology.

In reference to the host genus *Wurfbainia*, from which the holotype was isolated.

##### Holotype.

MHZU 23-0254.

##### Description.

***Saprobic*** on dead stem of *Wurfbainiavillosa*. ***Sexual morph***: undetermined. ***Asexual morph***: Colonies superficial on host substrate, effuse, hairy, gregarious, with numerous dark conidia on the substrate visible as black granular powder. ***Conidiophores*** 80–235 × 3–5.5 µm (av. 155 × 4.5 μm, n = 20), macronematous, mononematous, erect, simple, unbranched, straight or flexuous, subcylindrical, unevenly olivaceous brown, 1–3-septate, not constricted at the septa, smooth-walled to finely verruculose in the above half, thick-walled, with bulbous apices, bearing 5–8 conidiogenous cells at the tip, often intermixed with setiferous, flexuous, sterile filaments. ***Setae*** 230–390 × 3–6 µm (av. 305 × 4.5 μm, n = 20), arising from the basal stroma, adjacent to cells that give rise to fertile conidiophores, unbranched, straight, and subhyaline at base, mostly flexuous, olivaceous green, in above half, moderately thick-walled, smooth, septate, acute at apex. ***Conidiogenous cells*** 6–10 × 4–7 µm (av. 7.5 × 5.5 μm, n = 30), enteroblastic, monophialidic, discrete, determinate, terminal, elongate doliiform, pale to dark brown, smooth-walled, with a conspicuous collarette. ***Conidia*** 5–9 µm diam. (av. 7 μm, n = 30), acrogenous, solitary, dry, obovoid to subglobose, aseptate, hyaline, and smooth-walled when young, pale brown, mostly olivaceous to dark brown, verrucose to warty-surfaced at maturity.

##### Culture characteristics.

Colonies on PDA reaching 2 cm in two weeks at 28 ± 2 °C, medium dense, raised, sparse, filamentous, floccose to fluffy, velvety, filiform at margin, cream to pale brown from above; brown to pale luteous from reverse.

##### Material examined.

China • Guangdong Province, Yangchun City, Yongning Town (22.256185°N, 111.609037°E, 270 m), on dead stems of *Wurfbainiavillosa* (Lour.) Škorničk. & A.D. Poulsen. (Zingiberaceae), 10 April 2022, C.F. Liao & Y.H. Yang, YAM16 (MHZU 23-0254, holotype) • ex-type, ZHKUCC 23-1011 • *ibid*., living culture ZHKUCC 23-1012, and ZHKUCC 23-1013.

**Figure 2.**
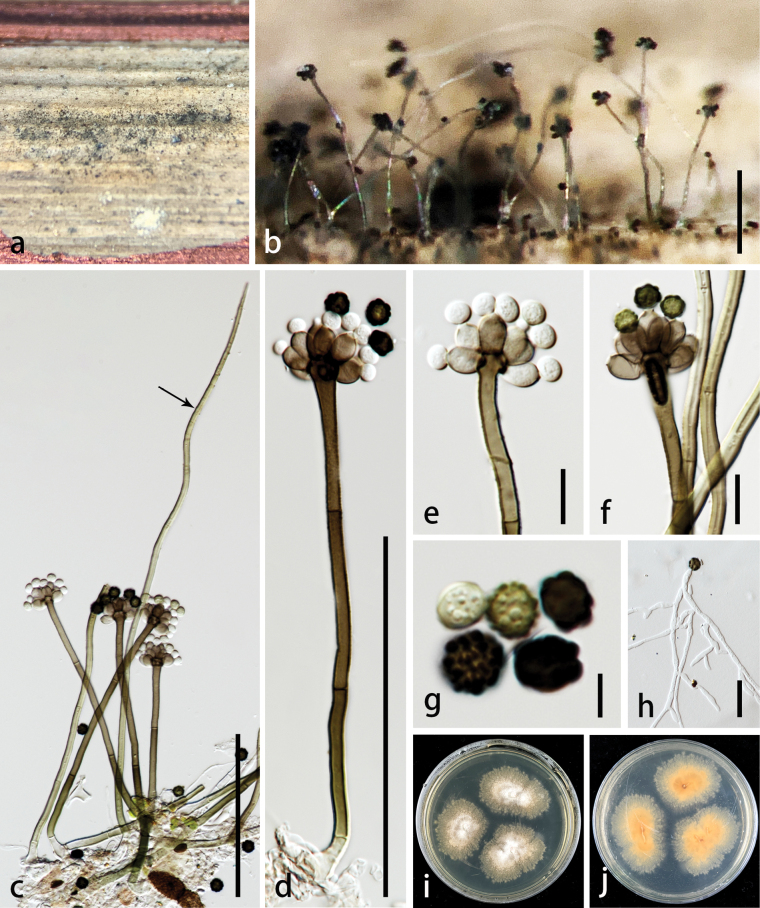
*Brevistachyswurfbainiae* (MHZU 23-0254, holotype) **a, b** colonies on the surface substrate **c, d** conidiophores, setae or conidiophore-like (arrow), conidiogenous cells, and conidia **e, f** conidiogenous cells with conidia **g** conidia **h** germinated conidium **i, j** colonies on PDA (front and below). Scale bars: 100 µm (**b–d**); 10 µm (**e, f**); 5 µm (**g**); 20 µm (**h**).

##### Notes.

*Brevistachyswurfbainiae* differs from other known species in the genus *Brevistachys* by having erect to flexuous, sterile, setiferous filaments intermixed with fertile conidiophores. *Brevistachyswurfbainiae* resembles *B.subsimplex* but differs from the latter in having slightly longer conidiophores with bulbous apices (80–235 × 3–5.5 µm vs. 80–200 (most frequently 100–140) × 3–5.5 µm) and shorter conidiogenous cells (6–10 × 4–7 µm vs. 8–13 × 4–6 µm). The conidiophores of *B.wurfbainiae* are 1–3-septate, while they are 2–6-septate in *B.subsimplex* (Deighton 1960). The phylogenetic analyses revealed that *B.wurfbainiae* (ZHKUCC 23-1011, ZHKUCC 23-1012, and ZHKUCC 23-1013) formed a separate branch from *B.subsimplex* (ex-type CBS 100155) with 75% ML bootstrap support and 0.93 BYPP (Fig. 1). Based on distinct morphology and phylogenetic support, we propose *B.wurfbainiae* as a new species.

#### ﻿ Nigrosynnema

Taxon classificationFungiHypocrealesStachybotryaceae

C.F. Liao, K.D. Hyde & Doilom gen. nov.

E293A5DB-224E-5817-8715-D4095987F34C

Index Fungorum: IF902006

Facesoffungi Number: FoF15744

##### Etymology.

The name refers to the characteristic black synnemata formed on natural substrate.

##### Description.

Saprobic on dead plant material. ***Sexual morph***: undetermined. ***Asexual morph*: *Conidiomata*** synnematous or sporodochial. ***Synnemata*** unbranched, subcylindrical, globose to subglobose head, robust at base, olivaceous brown to black, straight or curved in the upper portion, consisting of bundles of parallelly arranged, tightly compacted conidiophores. ***Sporodochia*** stromatic, superficial, scattered or gregarious, irregular, pulvinate, with white mycelium surrounding an olivaceous green mass of conidia. ***Conidiophores*** arising from basal stroma, macronematous, mononematous, septate, unbranched or branched, straight or flexuous, thin-walled, subcylindrical, olivaceous brown, verrucose, consisting of a stipe and a penicillately branched conidiogenous apparatus consisting of a whorl of primary branches, each terminating in number of conidiogenous cells. ***Conidiogenous cells*** enteroblastic, monophialidic, integrated, terminal, clavate to subcylindrical, hyaline to pale olivaceous brown, smooth, often verruculose at base, with a conspicuous collarette. ***Conidia*** solitary, fusiform to ellipsoidal, aseptate, initially hyaline, becoming olivaceous brown to dark brown, longitudinally striated at surface, with a distinct dark basal hilum.

##### Type species.

*Nigrosynnemaguangdongense* C.F. Liao, K.D. Hyde & Doilom

##### Notes.

*Nigrosynnema* resembles *Striaticonidium* in having fusiform to ellipsoidal conidia with longitudinal striations. However, it can be distinguished from *Striaticonidium* by having synnematous conidiomata, the absence of setae on the sporodochia, as well as support from molecular data. The synnematous conidiomata of *Nigrosynnema* are subcylindrical, flexuous, narrower towards the apex of the stipe, and robust at the base. The sporodochia are devoid of setae. However, in *Striaticonidium*, they are cylindrical to pyriform, broadened towards the apex, and have sporodochia covered by setae (Lombard et al. 2016). The blastn search of NCBI GenBank revealed that two strains of *Nigrosynnema*, ZHKUCC 23-1014 and ZHKUCC 23-1015, have sequence similarities of 98.37%, 91.73%, 89.70%, 89.04%, and 82.03% to the type species of *Striaticonidium* (*Stri.cinctum* CBS 932.69, ex-type) in LSU, ITS, *tub2*, *rpb2*, and *cmdA* sequence data, respectively. However, *tef1-α* sequence data of *Stri.cinctum* CBS 932.69 (ex-type) is unavailable in the NCBI database.

*Nigrosynnema* resembles *Virgatospora* described by Finley (1967) in having synnematous conidiomata, phialidic conidiogenous cells, and striated conidia. However, olivaceous brown to black synnemata in *Nigrosynnema* are subcylindrical, robust at the base, and narrower towards the apex of the stipe. The conidia in *Nigrosynnema* are aseptate, fusiform to ellipsoidal, and different from the septate, slightly curved conidia with a protuberant hilum of the type species of *Virgatospora*, *V.echinofibrosa*. *Nigrosynnema* can be distinguished from its closely related genera, as shown in Table 2.

**Table 2.** Morphological comparison of *Nigrosynnema* and its closely related genera.

Genera	Synnematous conidiomata		Sporodochial conidiomata	Conidiogenous cells		Conidia				Reference
	Shape	Color	Shape	Color	Shape	Color	Shape	Color	Number of septa	
*Nigrosynnema*	Subcylindrical, narrower towards the apex of the stipe with a robust base	Olivaceous brown to black	Irregular, with white mycelia surrounding an olivaceous green mass of conidia	Olivaceous brown to black	Enteroblastic, phialidic, monophialidic, subcylindrical	Mostly hyaline, sometimes pale olivaceous brown in the lower portion	Fusiform to ellipsoidal, longitudinally striated	Olivaceous brown to dark brown	Aseptate	This study
*Albosynnema*	Subcylindrical	Hyaline	-	-	Phialidic, cylindrical to subulate	Hyaline	Ellipsoidal to oblong	Blackish brown to black	3-septate	Morris (1967) and Bills et al. (1994)
*Didymostilbe*	Cylindrical, slightly thinned downwards, apex in a globose-hemispherical cap	White-gray	-	-	-	-	Short oblong-fusoid	Hyaline	0–1-septate	Saccardo and Saccardo (1906)
*Digitiseta*	-	-	Circular to ellipsoid	Hyaline	Penicillus biverticillate, phialides, cylindrical, finger-like, straight to slightly incurved inward the penicillus	Hyaline	Cylindrical to slightly asymmetrical	Hyaline to pale greenish	Aseptate	Gordillo and Decock (2017)
*Peethambara*	Cylindrical with a subglobose to oval head surmounted by conspicuous, slimy conidia	White	-	-	Phialide, mostly subcylindrical, sometimes wider in the middle than at either end	Hyaline	Ellipsoidal to limoniform, with mammiform basal and/or apical	Hyaline to subhyaline	1-septate	Subramanian and Bhat (1978)
*Striaticonidium*	Cylindrical to pyriform, broadening towards the apex	Marginal hyphae of synnemata olivaceous green	Oval to elongate or irregular with a white to grey setose fringe surrounding an olivaceous green to dark green slimy mass of conidia	Olivaceous green to mouse grey	Phialidic, clavate to cylindrical to subcylindrical	Hyaline	Fusiform to ellipsoidal, longitudinally striated	Olivaceous green to brown	Aseptate	Lombard et al. (2016)
*Virgatospora*	Cylindrical throughout the greater part, somewhat expanded at the apex and base	White or yellow at the base, yellow-black or blackish black at the apex	-	-	-	-	Campanulate, cylindrical, or allantoid to fusiform	Pale, olive to fuscous	Mature 3-(sometimes 2-or 4)-septate	Finley (1967)

The phylogenetic analyses supported that our two strains (ZHKUCC 23-1014 and ZHKUCC 23-1015) formed a distinct clade from other morphologically closely related taxa and constituted a well-supported clade related to *Digitiseta* with 94% ML and 1.00 BYPP statistical support. The main distinguishing morphological characteristic between the two genera is the absence of hypha-like setoid structures in *Nigrosynnema*, whereas *Digitiseta*, introduced by Gordillo and Decock (2017), has short apical branches and digitated hypha-like setoids. Additionally, the conidial shape is fusiform to ellipsoidal in *Nigrosynnema*, while they are cylindrical in *Digitiseta*.

Based on morphological and molecular evidence, we introduce a novel asexual genus, *Nigrosynnema*, characterized by olivaceous to black synnematous or sporodochial conidiomata that produce phialidic, aseptate conidia in black, slimy, glistening masses or heads. The conidia are fusiform to ellipsoidal, aseptate, longitudinally striated, and olivaceous brown to dark brown.

#### ﻿ Nigrosynnema guangdongense

Taxon classificationFungiHypocrealesStachybotryaceae

C.F. Liao, K.D. Hyde & Doilom sp. nov.

9BEEF1EB-45BC-5BF8-8321-B1616FC44D40

Index Fungorum: IF902007

Facesoffungi Number: FoF15746

Figs 3 , 4

##### Etymology.

The epithet “guangdongense” refers to the locality, Guangdong Province, China, where the holotype was collected.

##### Holotype.

MHZU 23-0255.

##### Description.

***Saprobic*** on dead stem of *Wurfbainiavillosa*. ***Sexual morph***: undetermined. Asexual morph: ***Synnemata*** on the natural substrate, 370–570 × 20–50 µm (av. 470 × 33 μm, n = 20), erect, unbranched, subcylindrical, with a robust base, narrowed towards fertile apex, olivaceous brown to black, straight or curved in the upper portion, consisting of parallelly arranged, tightly compacted conidiophores. ***Conidiophores*** 2–4 µm wide, subcylindrical, branched, olivaceous brown, slightly tapering towards the apex, verrucose. ***Conidiogenous cells*** 10.5–32.5 × 1.5–3 µm (av. 22 × 2.5 μm, n = 30), enteroblastic, monophialidic, discrete, terminal, subcylindrical, mostly hyaline, sometimes pale olivaceous brown in the lower portion, mostly smooth-walled in above half, often verruculose at below half, with a conspicuous collarette. ***Conidia*** 10–12.5 × 3–4.5 µm (av. 11.5 × 4 μm, n = 30), solitary, slimy, fusiform to ellipsoidal, aseptate, longitudinally striated, olivaceous brown to dark brown, guttulate, obtuse at both ends, with a distinct dark basal hilum.

**Figure 3.**
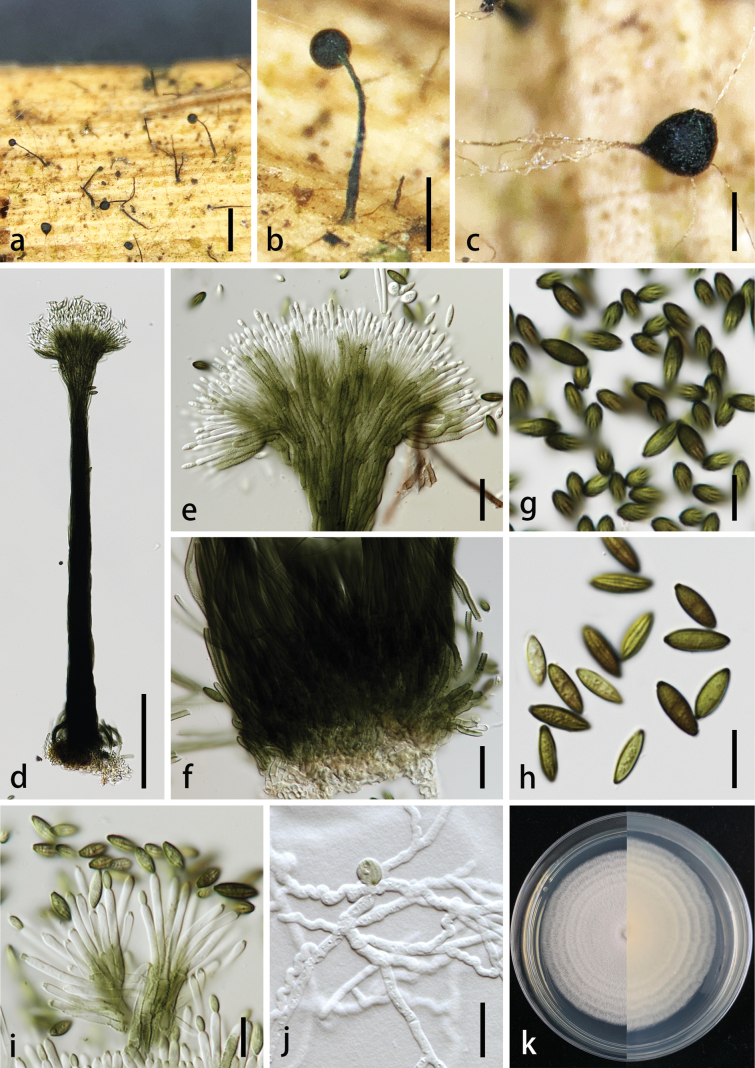
*Nigrosynnemaguangdongense* (MHZU 23-0255, holotype) on natural substrate **a–c** synnemata on substrate **d** synnema **e** top of synnema **f** base of synnema **g, h** conidia **i** conidiogenous cells with conidia **j** germinated conidium **k** colonies on PDA (front and below). Scale bars: 500 µm (**a**); 200 µm (**b**); 100 µm (**c, d**); 20 µm (**e, f**); 10 µm (**g–j**).

##### Culture characteristics.

Colonies on PDA reaching 4.5–6.5 cm in two weeks at 28 ± 2 °C, medium dense, flat or effuse, diffuse, rough, circular, filiform with curled, large circle in the middle becoming a wave and extends outward, cream from above; cream from the reverse. The spores produced on PDA after three weeks. ***Conidiomata*** 220–300 × 15–20 µm, sporodochial, superficial, scattered, irregular, with white mycelia surrounding an olivaceous green mass of conidia, with or without covering the slimy mass of conidia, without setae. ***Conidiophores*** arising from the basal stroma, consisting of a stipe and a penicillately branched conidiogenous apparatus; stipes unbranched or rarely branched, hyaline, septate, smooth, 10–30 × 2.5–3.5 µm (av. 18 × 3.0 μm, n = 20), conidiogenous apparatus consisting of a whorl of 2–5 primary branches, each terminating in 2–5 conidiogenous cells; primary branches, 1, 2-septate, smooth, unbranched, 8–20 × 2–6 µm, secondary branches, aseptate, smooth, unbranched, 6–20 × 2–5 µm. ***Conidiogenous cells*** 10–20 × 2–4 µm (av. 14 × 2.5 μm, n = 30), phialidic, terminal, with a conspicuous collarette, clavate to cylindrical, hyaline, smooth. ***Conidia*** 7–10 × 3–5 µm (av. 8.5 × 3.5 μm, n = 30), acrogenous, longitudinally striated, fusiform to ellipsoidal, aseptate, initially hyaline, becoming olivaceous green when mature.

**Figure 4.**
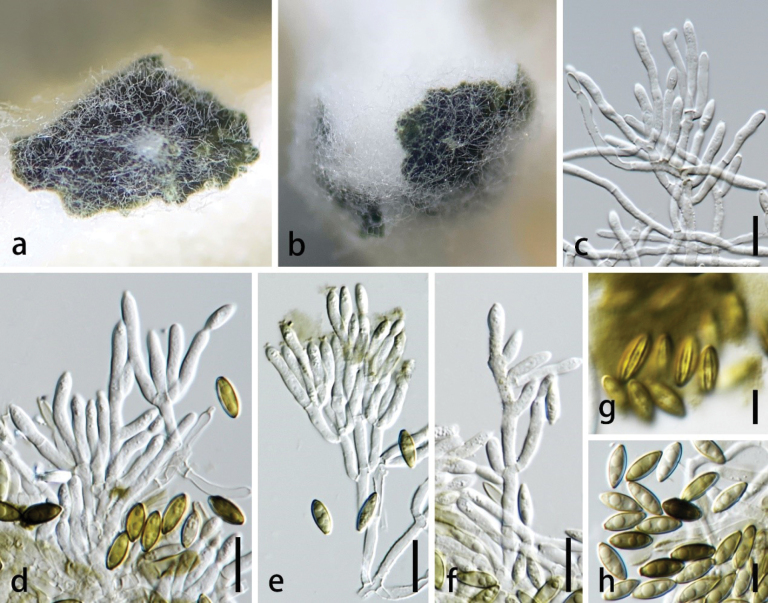
*Nigrosynnemaguangdongense* (MHZU 23-0255) on PDA after three weeks **a, b** conidial masses on pda **c–f** conidiophores, conidiogenous cells with conidia **g, h** conidia. Scale bars: 10 µm (**c–f**); 5 µm (**g, h**).

##### Material examined.

China • Guangdong Province, Yangchun City, Yongning Town (22.256185°N, 111.609037°E, 270 m), on dead stems of *Wurfbainiavillosa* (Lour.) Škorničk. & A.D. Poulsen. (Zingiberaceae), 10 April 2022, C.F. Liao & Y.H. Yang, YAM19 (MHZU 23-0255, holotype) • ex-type, ZHKUCC 23-1014 • *ibid*., living culture ZHKUCC 23-1015.

##### Notes.

*Nigrosynnemaguangdongense* is established here as the type species. It is similar to *Virgatosporanatarajanensis* described by D’Souza et al. (2002) in having synnematous conidiomata, with fusiform, aseptate, and striated conidia. However, *N.guangdongense* has verrucose, olivaceous, brown conidiophores, conidia with obtuse apices, and a distinct dark basal hilum, whereas *V.natarajanensis* has distinctly echinulate, subhyaline conidiophores that are narrower and smooth towards the apex, and conidia are rounded at both ends. Additionally, the conidiogenous cells of *V.natarajanensis* are occasionally found in the subterminal position, while they have not been observed in *N.guangdongense*. *Nigrosynnemaguangdongense* has longer conidiogenous cells (10.5–32.5 × 1.5–3 µm) compared to *V.natarajanensis* (18–25 × 1.5–3.5 μm).

*Nigrosynnemaguangdongense* and *Virgatosporaechinofibrosa* (the type species of *Virgatospora*) (Finley 1967) are similar in having synnematous conidiomata and striate, phialidic conidia. However, *N.guangdongense* has shorter synnemata (370–570 × 20–50 µm) than *V.echinofibrosa* (up to 1500 µm). Synnemata of *N.guangdongense* are robust at the base, narrower towards the apex of the stipe, and olivaceous brown to black, whereas they are simple or branched, sometimes proliferated, cylindrical throughout the greater part, somewhat broader at the apex and base, white or yellow at the base, yellow-black or blackish black at the apex in *V.echinofibrosa*.

Additionally, *N.guangdongense* has smaller (10–12.5 × 3–4.5), fusiform, aseptate conidia compared to ellipsoidal to limoniform, 3-(sometimes 2- or 4)-septate conidia (39–50 × 9–15 µm) of *V.echinofibrosa*.

#### ﻿ Nigrosynnema natarajanensis

Taxon classificationFungiHypocrealesStachybotryaceae

(D’Souza, S.K. Singh & Bhat) C.F. Liao, K.D. Hyde Doilom & Bhat comb. nov.

2515F308-9442-5A9D-AE98-AE53AD41F5BF

Index Fungorum: IF902010

##### Basionym.

*Virgatosporanatarajanensis* D’Souza, S.K. Singh & Bhat, Mycotaxon 82: 141 (2002).

##### Holotype.

IMI 386680.

##### Type information.

India • Middle Andaman Island, on dead leaves of *Calamusthwaitesii*, 15 December 2000, Rajiv Kumar, IMI386680 (holotype).

##### Description.

See D’Souza et al. (2002) on page 141.

##### Illustration.

See D’Souza et al. (2002) on page 140, Fig. 4a–d.

##### Notes.

D’Souza et al. (2002) introduced *Virgatosporanatarajanensis* based on the morphology and found it as a saprobe on dead leaves of *Calamusthwaitesii* from Middle Andaman Island, India. Although the DNA sequence data of *V.natarajanensis* is not available in NCBI, morphologically, it fits well within the generic concept of *Nigrosynnema* due to its synnematous conidiomata, phialidic conidiogenous cells, ellipsoidal to fusiform, aseptate conidia (amerosporous) with distinct longitudinal striations. The conidia in *V.echinofibrosa*, the type species of *Virgatospora*, are ellipsoidal to limoniform and 3-(sometimes 2- or 4)-septate (phragmosporous). Based on the morphological similarities between *V.natarajanensis* and *N.guangdongense* (the type species of *Nigrosynnema*), as well as support from molecular phylogenetic analyses of the type species of *Virgatospora*, we synonymize *V.natarajanensis* under *Nigrosynnema* as *N.natarajanensis*. However, it is desirable to obtain DNA sequence data from both the type specimen of *V.natarajanensis* and fresh collections to further support our proposal.

#### ﻿ Sirastachys guangdongensis

Taxon classificationFungiHypocrealesStachybotryaceae

C.F. Liao, K.D. Hyde & Doilom sp. nov.

B0810616-1D23-5049-8ADC-DDEE96115D8A

Index Fungorum: IF902009

Facesoffungi Number: FoF15747

Fig. 5

##### Etymology.

The epithet “guangdongensis” refers to the locality, Guangdong Province, China, where the holotype was collected.

##### Holotype.

MHZU 23-0250.

##### Description.

***Saprobic*** on dead stem of *Agavesisalana*. ***Sexual morph***: undetermined. ***Asexual morph***: Colonies superficial on host substrate, erect, gregarious, visible as numerous black conidial masses. ***Conidiophores*** 105–170 × 3.5–7 µm (av. 140 × 5.5 μm, n = 30), macronematous, mononematous, erect, simple, unbranched, straight or slightly flexuous, subcylindrical, hyaline, 1–5-septate, not constricted at septa, smooth-walled, or slightly verrucose, thick-walled, bearing 4–8 conidiogenous cells on the tip. ***Conidiogenous cells*** 6.5–12.5 × 4–5 µm (av. 10 × 4 μm, n = 30), enteroblastic, monophialidic, discrete, determinate, terminal, elongate doliiform to reniform, subhyaline to brown, smooth-walled, with a conspicuous collarette. ***Conidia*** 5–6 × 4–5 µm (av. 5.5 × 4 μm, n = 30), acrogenous, aggregating in slimy masses, obovoid, with a prominent hilum, aseptate, brown, pale olivaceous brown, black, smooth-walled.

**Figure 5.**
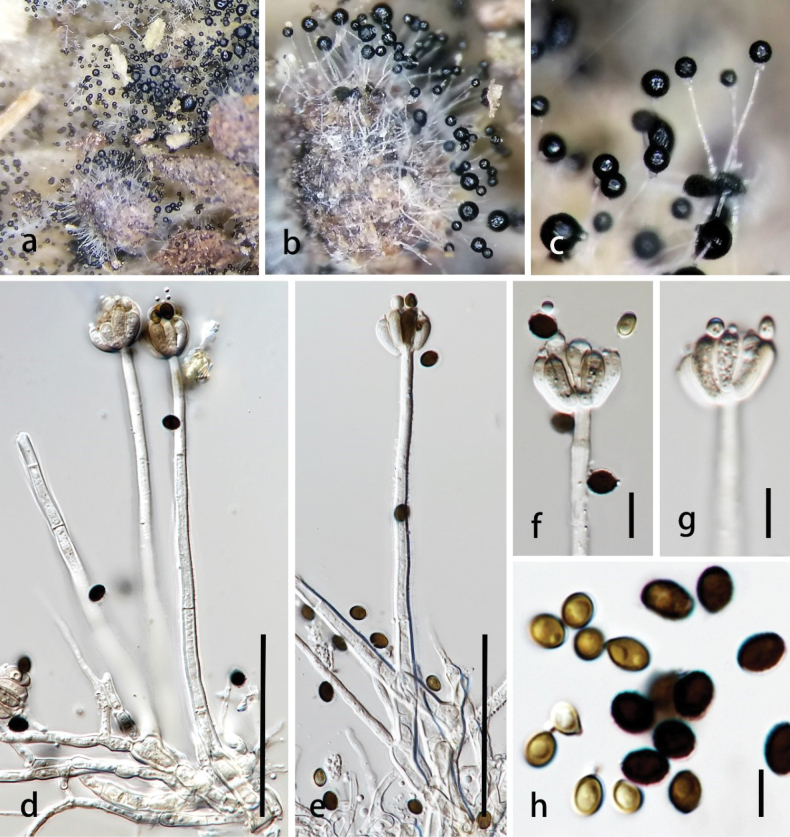
*Sirastachysguangdongensis* (MHZU 23-0250, holotype) **a–c** colonies on the surface substrate **d, e** conidiophores, conidiogenous cells with conidia **f, g** conidiogenous cells with conidia **h** conidia. Scale bars: 50 µm (**d, e**); 10 µm (**f, g**); 5 µm (**h**).

##### Culture characteristics.

Colonies on PDA reaching 5.5–6.0 cm in two weeks at 28 ± 2 °C, medium dense, flat, circular, cream from above; pale luteous from the reverse, with no pigmentation.

##### Material examined.

China • Guangdong Province, Guangzhou City, Zhongkai University of Agriculture and Engineering (23.10643°N, 113.28240°E, 20 m), on dead leaf of *Agavesisalana* Perr. ex Engelm. (Agavaceae), 17 November 2021, C.F. Liao & Y.H. Yang, JM02 (MHZU 23-0250, holotype) • ex-type, ZHKUCC 23-1003 • *ibid*., living culture ZHKUCC 23-1004.

##### Notes.

*Sirastachysguangdongensis* resembles *Si.pandicola* and *Si.Phaeospora* that were described by Lombard et al. (2016). However, the former can be distinguished by the size of the conidiophores and conidia as well as other conidiophore characteristics. *Sirastachysguangdongensis* has longer conidiophores (105–170 µm) than those of *Si.pandicola* (55–75 µm) and *Si.phaeospora* (40–65 µm). *Sirastachysguangdongensis* has larger conidia (5–6 × 4–5 µm) than *Si.pandicola* (3–4 × 2–3 µm) and *Si.phaeospora* (4–5 × 2–3 µm). Conidiophores of *Si.guangdongensis* are 1–5-septate, while they are 1–3-septate in *Si.pandicola* and 1–2(–3)-septate in *Si.phaeospora*. Branched conidiophores are observed in *Si.phaeospora* (Lombard et al. 2016), while they are unbranched in *Si.guangdongensis*. The phylogenetic analyses supported *Si.guangdongensis* as a distinct species from other *Sirastachys* species and showed that *Si.guangdongensis* (ZHKUCC 23-1003 and ZHKUCC 23-1004) formed a distinct branch and sister to *Si.phaeospora* (ex-type CBS 100155) with 99% ML bootstrap support and 1.00 BYPP (Fig. 1). Based on distinct morphological and molecular evidence, we propose *Sirastachysguangdongensis* as a novel species.

#### ﻿ Stachybotrys microsporus

Taxon classificationFungiHypocrealesStachybotryaceae

(B.L. Mathur & Sankhla) S.C. Jong & E.E. Davis [as ‘microspora’]

3CE5110C-40ED-50AA-A7D2-5A11DF12B5F6

Index Fungorum: IF627002

Facesoffungi Number: FoF09372

Fig. 6

##### Description.

***Saprobic*** on dead leaf of *Agavesisalana*. ***Sexual morph***: undetermined. ***Asexual morph***: Colonies superficial on host substrate, gregarious, visible as numerous black conidial masses. ***Conidiophores*** 35–70 × 2.5–5 µm (av. 48 × 4 μm, n = 30), macronematous, mononematous, irregularly or sympodially branched, straight or flexuous, subcylindrical, hyaline, becoming pale olivaceous brown in the above half, 1–3-septate, not constricted at the septa, smooth-walled, slightly rough-walled in the subterminal region, thick-walled, bearing 3–9 conidiogenous cells on the tip. ***Conidiogenous cells*** 6–10 × 4–6 µm (av. 8 × 5 μm, n = 30), enteroblastic, monophialidic, discrete, determinate, terminal, obovoid, sub-hyaline to pale olivaceous brown, smooth-walled. ***Conidia*** 5–7 µm diam. (av. 6 μm, n = 30), aggregating in slimy masses, globose, subglobose, aseptate, olivaceous brown to black, rough-walled, verrucose.

**Figure 6.**
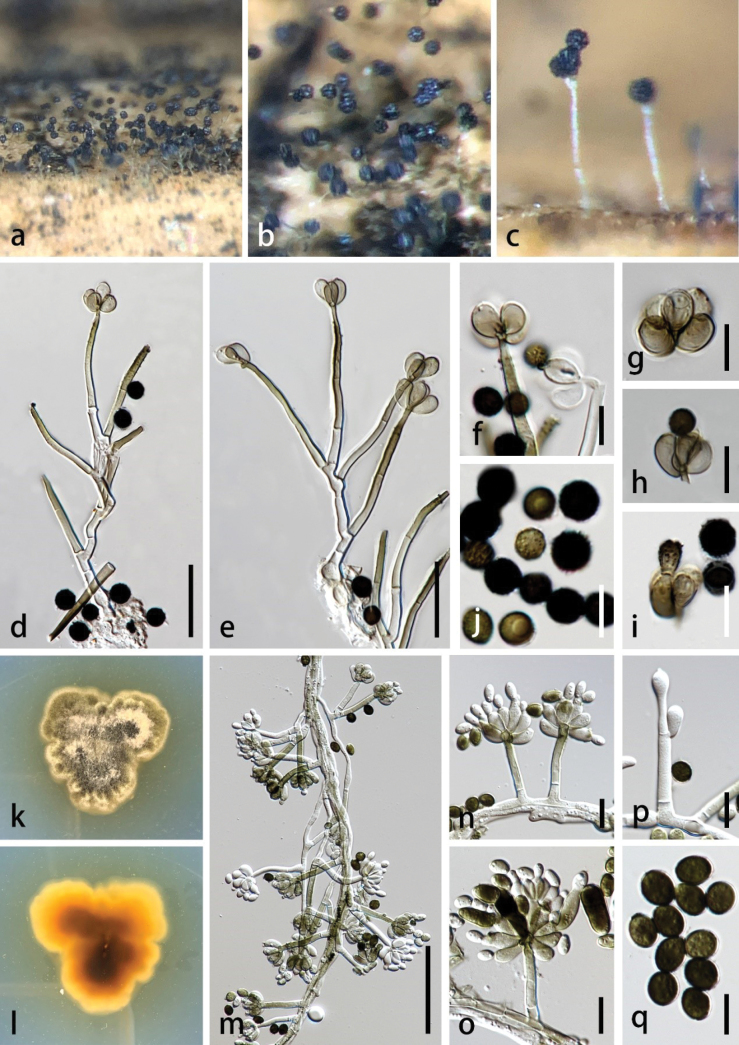
*Stachybotrysmicrosporus* (MHZU 23-0252, new host record) **a–c** colonies on the surface substrate **d, e** conidiophores **f–i** conidiogenous cells **j** conidia **k** colonies on pda (front) **l** colony on pda (below) **m** mycelium, conidiophores, and conidiogenous cells with conidia **n–p** conidiogenous cells with conidia **q** conidia. Scale bars: 20 µm (**d, e**); 50 µm (**f–j, m**); 10 µm (**n–q**).

##### Culture characteristics.

Colonies on PDA reaching 2.5–3.0 cm in two weeks at 28 ± 2 °C, medium dense, raised, flat, floccose to fluffy, velvety, irregular edge, gold brown at the center, pale brown, with conidiophores forming on the surface of the medium, carrying slimy olivaceous green from above; brown to pale luteous from the reverse. The conidia producing on PDA after three weeks: Conidiophores 30–80 × 3.5–5.5 µm (av. 46 × 4 μm, n = 30), most similar with the above description, 0–2-septate, unbranched or branched, bearing 2–10 conidiogenous cells at the tip. Conidiogenous cells 9–17 × 4.5–7.5 µm (av. 12 × 5.5 μm, n = 30), most similar to those on natural substrate. Conidia 6.5–10 × 3–6.5 µm (av. 8 × 5 μm, n = 30), often aggregated as large, slimy, glistening, blackheads, initially hyaline to olivaceous green, oblong, obovoid to subglobose, becoming black-brown, globose, smooth to verruculose.

##### Material examined.

China • Guangdong Province, Guangzhou City, Zhongkai University of Agriculture and Engineering (23.10643°N, 113.28240°E, 20 m), on dead leaf of *Agavesisalana* Perr. ex Engelm. (Agavaceae), 17 November 2021, C.F. Liao & Y.H. Yang, SR09A (MHZU 23-0252, new host record) • living culture, ZHKUCC 23-1007 • *ibid*., living culture ZHKUCC 23-1008.

##### Known distribution.

• Canada, Cuba, India, Nigeria, and Pakistan (Ellis 1971; Jong and Davis 1976); • China (this study); Japan (Iwama et al. 2022); • New Guinea; Zaria (Jong and Davis 1976); • Thailand (Lin et al. 2016; Samarakoon et al. 2021); • Sudan (Lombard et al. 2016).

##### Known hosts/substrates.

*Agavesisalana* (this study), *Arachishypogaearhizosphere*, soil (Jong and Davis 1976), *Castanopsiscuspidata* var. sieboldii (Iwama et al. 2022), dead plants, paper, seeds, and textiles (Ellis 1971), decaying shrubs, wood (Lin et al. 2016), *Musa* sp. (Samarakoon et al. 2021), soil in *Mangifera* field (Lombard et al. 2016).

##### Notes.

*Stachybotrysmicrosporus* (ZHKUCC 23-1007, ZHKUCC 23-1008) formed a subclade with the type and other strains of *St.microsporus* with 100% ML and 1.00 BYPP (Fig. 1). Our collection has similar morphs to *St.microsporus* described by Jong and Davis (1976), Lin et al. (2016), and Samarakoon et al. (2021) by having irregularly branched conidiophores with tapering apices, monophialidic, discrete conidiogenous cells, unicellular, globose, roughened, and black conidia. *Stachybotrysmicrosporus* has been reported from forest soil in New Guinea (Jong and Davis 1976), on decaying wood in Thailand (Jong and Davis 1976), and on dead leaf petiole of *Musa* sp. in Thailand (Samarakoon et al. 2021). We report *St.microsporus* here as a new host record on *Agavesisalana* in China.

## ﻿Discussion

Species of Stachybotryaceae have primarily been collected from soil and dead plant tissues. For example, *Albifimbriaterrestris* was isolated from soil in mopane woodlands in Namibia and unidentified dead hardwood in the USA, while *Cymostachysfabispora* was obtained from decaying leaf material in Cuba and *Aloeferox* in Tanzania (Lombard et al. 2016). In China, most taxa in this family have been reported from the soil, including *Stachybotryspallescens*, *St.subcylindrospora*, *St.subreniformis*, and *St.subcylindrospora* (Jiang and Zhang 2009; Li and Jiang 2011; Jie et al. 2012). However, some species were also found in diseased plants and dead plant tissues. *Paramyrotheciumroridum* (formerly known as *Myrotheciumroridum*) has been identified as a pathogen causing leaf spot on *Abutilonmegapotamicum* and *Zantedeschiaaethiopica* plants from China (Li et al. 2014; Ben et al. 2015). *Myxosporaaptrootii* was isolated from leaf litter in Hong Kong, China (Lombard et al. 2016). In this study, *Brevistachyswurfbainiae* and *Nigrosynnemaguangdongense* were isolated from dead stems of *Wurfbainiavillosa*, while *Sirastachysguangdongensis* and *Stachybotrysmicrosporus* were obtained from dead leaves of *Agavesisalana* in Guangdong, China. The present study contributes to the taxonomic and phylogenetic study of Stachybotryaceae by introducing a novel genus and two new species, along with the documentation of one newly recorded species from China.

The new genus, *Nigrosynnema*, is phylogenetically related to but distinct from *Digitiseta* and *Striaticonidium*. These taxa are classified into distinct genera based on differences in their asexual morphs, as detailed in the notes under *Nigrosynnema*. *Nigrosynnema* is also similar to *Peethambara* in having synnematous, erect conidiomata, unbranched or branched, septate, smooth conidiophores with phialidic conidiogenous cells (Subramanian and Bhat 1978). However, *Peethambara* has elongate or elongate-fusiform or broad-fusiform, hyaline, thick-walled, 1-septate conidia in green slimy masses, which are mostly widest in the middle, sometimes above or below the middle, and with smoothly rounded mamilla at the base (Subramanian and Bhat 1978; Lombard et al. 2016). Furthermore, *Nigrosynnema* can be distinguished from other asexual genera in Stachybotryaceae by its black, subcylindrical synnema that tapers towards the apex (Table 2) and the support from robust phylogeny. Additionally, our study found that *Nigrosynnemaguangdongense*, the type species of this novel genus, produced synnematous conidiomata on the natural host substrates and sporodochial conidiomata on PDA. Synnemata observed on the natural substrate are erect, with a robust base, subcylindrical, narrower towards the apex of the stipe, and olivaceous brown to black in color. Synnemata appear slender, straight, or curved in the upper portion and consist of bundles of parallelly arranged, tightly compacted conidiophores. On the other hand, sporodochial conidiomata appeared on PDA, producing superficially scattered irregular structures on pulvinate conidiophores and surrounded by white mycelia and crowned by an olivaceous green mass of conidia with or without a slimy covering.

This study proposes *Sirastachysguangdongensis* as a novel species in the family Stachybotryaceae, represented by the strains ZHKUCC 23-1003 and ZHKUCC 23-1004. It is noted that Lombard et al. (2016) designated CBS 100155 as the ex-type of *Sirastachysphaeospora* and identified additional strains (CBS 136167, CBS 136185, CPC 16092, CPC 16093, and CBS 253.75) as *Si.phaeospora*. However, their phylogenetic analyses did not support the formation of a well-defined monophyletic lineage for these additional strains with the ex-type. Although the materials examined included CBS 136167, CBS 136185, and CPC 16092, only ex-type (CBS100155) was used to illustrate the morphological characteristics of *Si.phaeospora*. Therefore, it is recommended to confirm the taxonomic identification of these additional strains and determine whether they are congeneric with the ex-type of *Si.phaeospora*. In this study, we accepted only CBS 100155 as an authentic strain of *Si.phaeospora* for inclusion in our phylogenetic tree, in which our new collections (ZHKUCC 23-1003 and ZHKUCC 23-1004) formed a distinct branch sister to *Si.phaeospora* (ex-type CBS100155) with 99% ML bootstrap support and 1.00 BYPP (Fig. 1). The morphological characteristics observed in our collection differ from those described for *Si.phaeospora* based on characteristics of conidiophores, as well as the size of conidiophores and conidia, as described in the notes under *Si.guangdongensis*.

The taxonomic placement and phylogenetic relationship of many species, such as *Myrotheciumatrocarneum*, *Stachybotrysasperulus*, *St.atrogriseus*, *St.atrus*, *St.clitoriae*, and *St.verrucosus* in Stachybotryaceae, remain unclear due to a lack of DNA sequence data of ex-type strains and fresh collections. Furthermore, many species in Stachybotryaceae are limited to ITS and LSU sequence data. There is a scarcity of reliable phylogenetic markers (e.g., *cmdA*, *rpb2*, *tef1*-α, and *tub2*) to identify the phylogenetic status within Stachybotryaceae accurately. Future taxonomic studies in this family should incorporate multi-locus genes, such as *cmdA*, ITS, LSU, *rpb2*, *tef1*-α, and *tub2*, along with morphological characteristics and other polyphasic approaches (e.g., physiology and secondary metabolites), while also considering hosts and their distribution to enhance our understanding in this issue (Maharachchikumbura et al. 2021). Additionally, the specimen of the type species should be revisited, and epitypification is needed to confirm their taxonomic placement.

## Supplementary Material

XML Treatment for Brevistachys wurfbainiae

XML Treatment for Nigrosynnema

XML Treatment for Nigrosynnema guangdongense

XML Treatment for Nigrosynnema natarajanensis

XML Treatment for Sirastachys guangdongensis

XML Treatment for Stachybotrys microsporus

## ﻿Additional information

### Author ORCIDs

Chunfang Liao https://orcid.org/0000-0002-6309-1101

Mingkwan Doilom https://orcid.org/0000-0001-6167-3637

D. Jayarama Bhat https://orcid.org/0000-0002-3800-5910

Kandawatte Wedaralalage Thilini Chethana https://orcid.org/0000-0002-5816-9269

Khanobporn Tangtrakulwanich https://orcid.org/0009-0002-7081-618X

Yunhui Yang https://orcid.org/0000-0002-0326-1471

Fatimah Al-Otibi https://orcid.org/0000-0003-3629-5755

Kevin D. Hyde https://orcid.org/0000-0002-2191-0762

Wei Dong https://orcid.org/0000-0003-4991-1529

### Data availability

All of the data that support the findings of this study are available in the main text.
